# Piriformis preservation in total hip arthroplasty: do we have a new concept? An update on anatomy, function and clinical outcomes

**DOI:** 10.1530/EOR-2023-0184

**Published:** 2025-05-05

**Authors:** Eustathios Kenanidis, Eleni Gkoura, Eleni Tsamoura, Zakareya Gamie, Peter Sculco, Eleftherios Tsiridis

**Affiliations:** ^1^ Academic Orthopaedic Department, Aristotle University Medical School, General Hospital Papageorgiou, Thessaloniki, Greece; ^2^ Centre of Orthopaedic and Regenerative Medicine (CORE), Center for Interdisciplinary Research and Innovation (CIRI)-Aristotle University of Thessaloniki (AUTH), Balkan Center, Thessaloniki, Greece; ^3^ Department of Orthopedic Surgery, Hospital for Special Surgery, New York, New York, USA

**Keywords:** piriformis, piriformis muscle, muscle-preserving, total hip arthroplasty, mini posterior approach

## Abstract

- The piriformis muscle (PM) is important for posture and preventing falls. It is a key landmark for hip surgery. The PM function is reported to be increasingly important for improving total hip arthroplasty (THA) outcomes and reducing complications. This scoping review aims to map and summarize the literature on the anatomy and function of the PM and the outcomes of clinical studies on THA preserving the PM to improve readers’ understanding and identify areas for further research.
- A scoping review following the PRISMA guidelines was conducted using PubMed and Scopus from their inception until June 2023. We used the search term ‘piriformis’ or ‘PM’ to include all PM-related studies. Two independent reviewers screened abstracts and full texts to select key aspects of PM anatomy and function and the main clinical THA studies reporting outcomes on PM preservation.
- Fifty-seven studies published between 1980 and 2023 met our inclusion criteria. During hip surgery, the PM anatomy, including its origin and insertion, muscle belly, and relation to other short hip rotators and the sciatic nerve, can vary greatly, making it difficult to recognize.
- The current literature on PM-preserving THA and hemiarthroplasty clinical studies is limited. It suggests potential benefits in terms of hip stability, dislocation risk, and functional outcomes compared to no PM preservation in short-term follow-up.
- Identifying and preserving the PM during hip surgery may be difficult due to its variable anatomy and its relation to surrounding structures. Although the literature supporting PM preservation potentially indicates better outcomes, further high-level research studies are needed.

## Introduction

The piriformis muscle (PM) has a pyramidal shape and originates from the anterior surface of the sacrum. It passes through the greater sciatic foramen and attaches to the femur’s greater trochanter (GT) (1). This muscle is part of the short external hip rotators (SHERs), helping also to abduct and stabilize the hip joint. Various PM and tendon variations have been reported, ranging from agenesis or a duplicate muscle to complex relationships with the other SHERs and the sciatic nerve.

The PM is uniquely found only in hominoids and not in other vertebrates (2). It is crucial in maintaining posture, stabilizing the body in a standing or walking position and preventing falls (1). The PM is also highly significant as an essential anatomical landmark in the deep gluteal region for various medical procedures, including ultrasound scans, nerve blocks, acetabular fracture repair and total hip arthroplasty (THA) (3, 4). Understanding the PM anatomical variations when performing hip surgery or diagnosing pathology is essential. During the standard posterior THA approach, the PM is tenotomized to gain access to the hip joint and is reinserted into the GT at the end of the procedure. However, several piriformis-preserving posterior hip approaches have been reported to improve hip stability, postoperative outcomes and patient pain relief (5, 6).

Although the PM holds significant clinical value, no comprehensive review of its anatomy, function and relation to surrounding structures exists. Given the growing interest in the PM, we recognize the need to systematically gather the existing literature for this review. This scoping review aims to map and summarize the literature on the anatomy and function of the PM and the outcomes of clinical studies on THA preserving the PM to improve readers’ understanding and identify areas for further research.

## Methodology

Our scoping review followed the Preferred Reporting Items for Systematic Reviews and Meta-Analyses (PRISMA) guidelines. This scoping review protocol was not required to be pre-registered in the international prospective register of systematic reviews (PROSPERO).

### Search strategy

Our research team conducted a comprehensive data search from April to June 2023 to find relevant studies. We searched PubMed and Scopus from their inception until June 2023 and checked the reference lists of the included studies and the library of our medical university. Our search string was ‘(piriformis [ti] OR piriformis [tw] OR PM [ti] OR PM [tw])’. At first, our search was narrowed to include all PM-related studies.

### Eligibility criteria

All types of studies were included in this research, including comparative and non-comparative, retrospective and prospective studies, controlled trials, book chapters, reviews, systematic reviews and meta-analyses. Cadaveric and non-cadaveric studies, case series, case reports and reviews on piriformis syndrome were also included to gain valuable knowledge about PM anatomical variations. Studies were excluded if they were written in a non-English language or had no full text available. In addition, systematic reviews with a similar context to the present systematic review were also excluded.

### Outcomes

Our main objective was to gather the existing data on the anatomy, function and relationship to other anatomical structures (such as the sciatic nerve and SHERs) and anatomical PM variations. Our secondary goal was to map and summarize the outcomes of clinical studies on THA-preserving PM to improve readers’ understanding and identify areas for further research.

### Study selection and extraction of data

The articles were assessed by two independent researchers, EG and ET. They read through the titles and abstracts, selecting relevant reports. Afterward, the eligible studies were reevaluated and duplicates were excluded. A senior author resolved any discrepancies between the two authors. During their evaluation, EG and ET extracted data about the muscle’s anatomy from each study, including details such as muscle belly, tendon, origin, insertion, function and relation to other anatomic structures such as the sciatic nerve and SHERs and clinical studies on THA-preserving PM. They also noted any anatomic variations.

## Results

**Figure 1:**
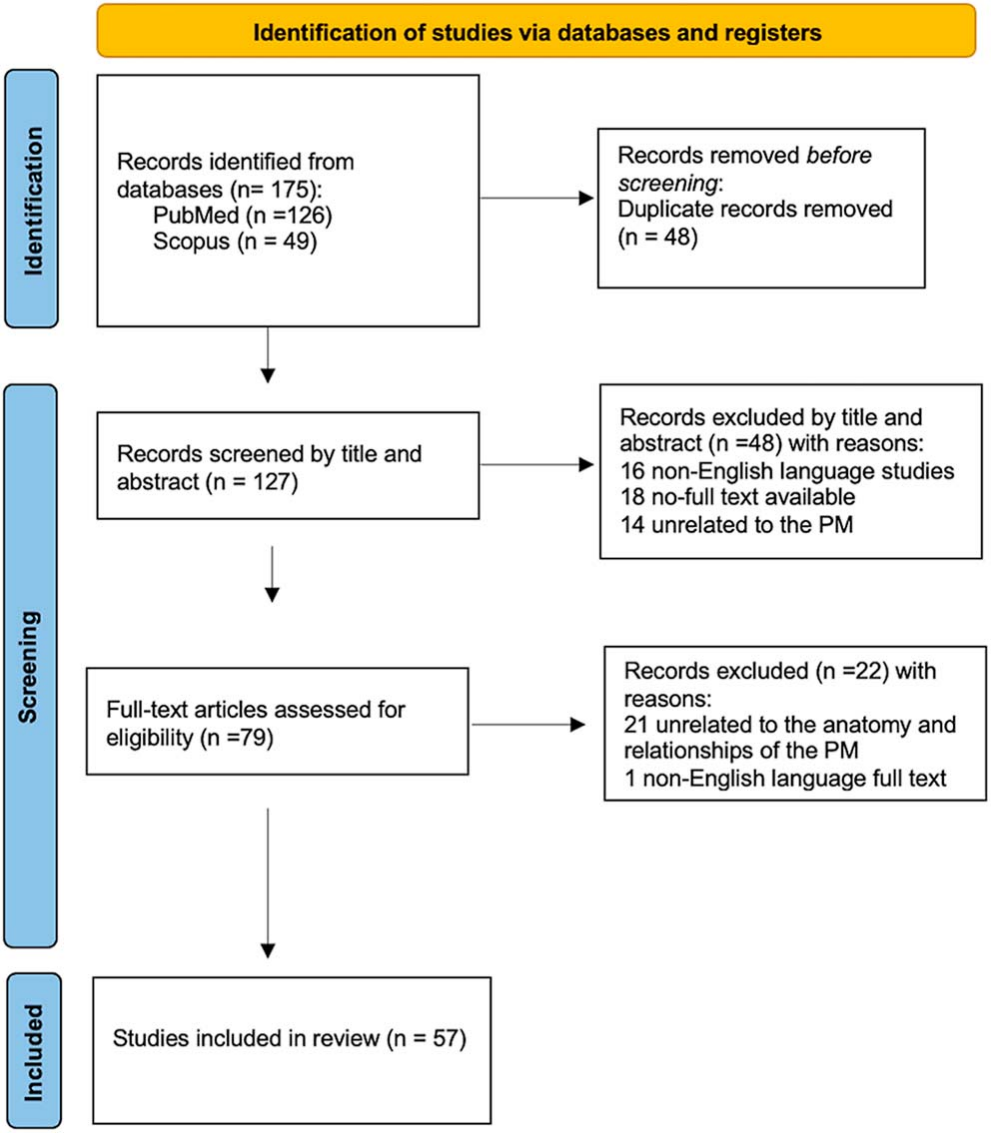
Flow diagram of search strategy.

The initial electronic search retrieved 175 studies. After removing 48 duplicate studies, we reviewed the titles and abstracts of the remaining studies. Based on our predefined inclusion and exclusion criteria, 48 records were excluded and the full text of the remaining 79 papers was examined. Ultimately, our scoping review encompassed 57 studies (1, 2, 5, 6, 7, 8, 9, 10, 11, 12, 13, 14, 15, 16, 17, 18, 19, 20, 21, 22, 23, 24, 25, 26, 27, 28, 29, 30, 31, 32, 33, 34, 35, 36, 37, 38, 39, 40, 41, 42, 43, 44, 45, 46, 47, 48, 49, 50, 51, 52, 53, 54, 55, 56, 57, 58, 59, 60). The study’s flowchart is presented in Fig. 1.

We reviewed studies published between 1937 and 2022, including nine reviews (7, 11, 12, 27, 38, 40, 43, 44, 47), 15 cadaveric studies (13, 14, 15, 23, 24, 25, 26, 28, 29, 32, 33, 34, 35, 36, 39), 11 case reports (2, 8, 9, 16, 17, 18, 19, 20, 31, 41, 45, 55), five retrospective MRI studies (10, 21, 22, 59, 60), two books (1, 37), two systematic reviews (30, 42), three randomized control trials (46, 50, 52), three comparative studies (6, 56, 58), one observational study (53) and six case series (5, 48, 49, 51, 54, 57).

### Origin, course and insertion

**Figure 2:**
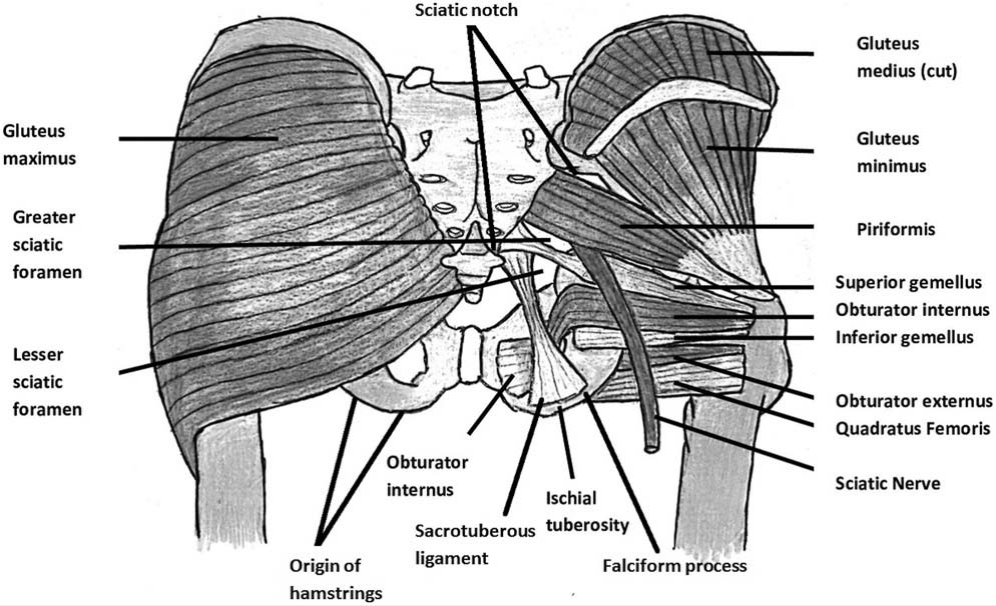
Schematic representation of the main anatomical structures of the subgluteal region.

The PM is a pyramid-shaped muscle that forms part of the SHERs. It lies beneath the gluteus maximus and below the gluteus medius (GMed), bordering the gluteus minimus in the subgluteal space. This area is restricted laterally by the linea aspera, medially by the falciform fascia and sacrotuberous ligament, superiorly by the sciatic notch and inferiorly by the origin of the hamstrings (7). Along with the PM, other SHERs such as the superior gemellus, obturator internus (OI), inferior gemellus and quadratus femoris are also present in the subgluteal space (Fig. 2).

#### Origin

PM origin has been reported in various anatomical regions. The muscle may arise from the anterior surface of the first to fourth sacral foramina (8), the second to fourth sacral foramina (7, 9), the second to third sacral foramina (10, 11), as well as the anterolateral sacrum and the top iliac part (1). Along with these regions, muscle fibers may also originate from the spinal portion of the gluteal muscles, the sacroiliac capsule and the sacrotuberous ligament (12).

#### Course

The PM extends laterally, passing through the greater sciatic foramen before moving anteriorly and inferiorly to attach to the GT. It divides the greater sciatic foramen into superior and inferior parts as it passes (Fig. 2). The superior part provides a pathway for the superior gluteal nerve and artery, while the inferior part is crossed by various nerves and blood vessels, including the inferior gluteal nerve and artery, internal pudendal vessels, sciatic nerve, posterior femoral cutaneous and pudendal nerves, and the nerve to the OI and quadratus femoris.

#### Insertion point

There is a disagreement among scientists regarding the precise PM tendon insertion point. Previously, it was thought to be the ‘piriformis fossa’ on the medial side of the superior GT aspect (1). However, recent studies have shown that this depression is where the obturator externus is inserted and unrelated to the PM (13, 14). The piriformis fossa was considered the optimal entry point for femoral nail insertion. However, a study revealed that only 24% of the femurs had the fossa aligned with the femoral shaft direction (13).

**Figure 3:**
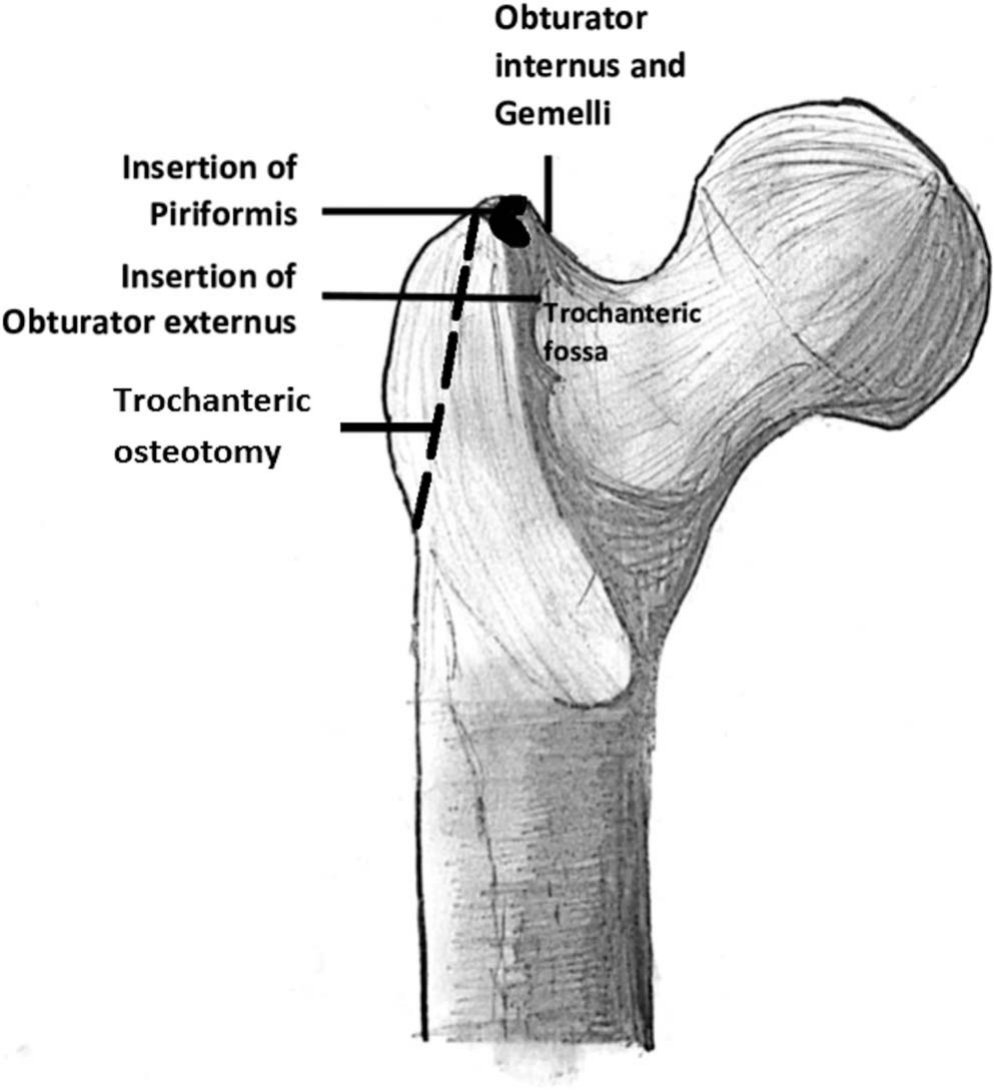
Typical insertion sites of short external hip rotators at the superior posterior femoral surface.

The piriformis tendon usually attaches on the medial upper and posterior side of the GT upper part and slightly laterally to the OI tendon insertion point (15). It can also attach to the hip joint fibrous capsule. According to a study, the femoral PM attachment has a horseshoe shape with distinct anterior and posterior margins (15) (Fig. 3). This study measured the distance between the front and back edges of the piriformis tendon insertion and the far end of the GT as a percentage of the GT length. The average distance for the anterior margin was 63%, while the average distance for the posterior margin was 43%. Similarly, the OI insertion measurements were 73 and 55% for the anterior and posterior margins, respectively. The study concluded that the attachment of these muscles may be more than one-third of the way along the GT, which suggests that current THA osteotomies may not include these SHERs (5, 15).

### Anatomical variations

Various anatomical PM variations have been documented in the literature, including absence or agenesis (2, 8, 16, 17, 18), accessory muscle (19, 20) or double PM (21). According to a recent MRI study, the PM size varied from 0.8 to 3.2 cm, with an average size of 1.9 cm. Furthermore, 19% of patients exhibited more than 3 mm of asymmetry in PM size, with the most remarkable asymmetry measuring up to 8 mm (22). Another study revealed that 70% of patients exhibit a pear-shaped PM morphology (23). Furthermore, in 20% of cases, the PM is separated into two parts, with the common peroneal nerve passing through them. In the remaining 10%, the PM is fused with the GMed muscle, causing the superior gluteal nerve and vessels to run between the fibers of the fused muscles (23).

The PM is characterized by having multiple muscle bellies, with the two-bellied muscle being the most common type (8). Three distinct muscle types exist depending on the muscle bellies’ degree of fusion. Type A (63.39%) features a shorter upper muscle belly further from the insertion point, while type B (35.71%) has a longer upper muscle belly closer to the insertion point. The rarest type is type C (0.9%), where both bellies merge at the same level. Females may also exhibit a difference between the right and left sides, with the right upper muscle belly ending closer to the origin than the left (24).

### Tendon

Cadaveric studies have revealed interesting findings on the PM musculotendinous junction. The junction is roughly 50 mm from the insertion point (15) with the tendon measuring 3–9 mm in diameter at this level (24). Notably, the tendon size varies at the insertion point, with males having an average diameter of 15.6 mm in the anteroposterior plane while females have an average of 14.3 mm (25). Besides, the right side’s tendon is usually thicker than the left (15).

A cadaveric study of 15 femora revealed an uncommon insertion site for the PM tendon. In this case, the tendon was divided into two parts, each with a slightly different insertion point (14). Both tendon sections were connected to the GT, but one was located more towards the posterior than the other. Sometimes, the PM tendon may combine with tendons coming from the other SHERs. A study of 112 cadavers examined variations in the PM tendon compared to the other SHERs and categorized them into four groups (24) presented in Table 1. Another study involving 29 femur cadavers revealed varying insertion patterns for the PM and OI tendons (15). The PM tendon consistently crosses over the OI tendon at an average angle of 42°. The research identified four distinct categories based on the tendon insertion patterns, thoroughly outlined in Table 2.

**Table 1:** Anatomic variations of piriformis tendon insertion in relation to the insertion of the other short external rotator muscles. Data derived from Windisch *et al.* (24).

Type	Pattern	Attachment site	Frequency
1	Single tendon insertion	GT	53.57%
2	Fusion with SG and OI tendons	The common tendon attaches to GT superior to the trochanteric fossa	29.46%
3	Fusion with GM and OI tendons	The common tendon attaches medially to GT superior to the trochanteric fossa	13.39%
4	Merging with GM fibers	GT	3.57%

SG, superior gemellus; OI, obturator internus; GM, Gluteus medius; GT, greater trochanter.

**Table 2:** Anatomic variations of piriformis (PF) tendon insertion in relation to the insertion of the OI.

Type	Angle pattern between tendons/description	Frequency
1	Perpendicular to each other	55%
	The posterior margin of OI overlaps with the anterior margin of the PF tendon	
2	<90 degrees	17%
	a. PF tendon is flattened on the horizontal plane	
	b. The overlap is also greater than in type I	
	c. The crossing angle is the biggest out of all types	
3	PF tendon is directly superior to the OI tendon	17%
4	The tendons fuse and attach as a common or separate tendons	10%

OI, obturator internus.

### Relationship with SHERs

The group of SHERs includes the PM, the gemelli, the OI and externus and the quadratus femoris (Fig. 2). The PM is located above the other SHERs. When comparing the length of the SHERs, a study found that the PM was the second largest muscle (mean total length: 14 cm) behind the OI (mean full length: 16 cm) (26). Sometimes, the OI tendon merges with the other SHER tendons such as the gemelli and PM. The length of the conjoint tendon of the OI and PM muscles is reported to be between 0.5 and 2 cm (26). It is separated from the GMed muscle by a fat pad. Although the tendon fibers are merged, they typically follow the original muscle course with the PM fibers lying anteriorly and superiorly. Before inserting on the GT, the conjoint tendon also connects to the joint capsule, the GM and the OI muscle with connections greater than 1 cm (26).

### Relationship between the PM and the sciatic nerve

**Figure 4:**
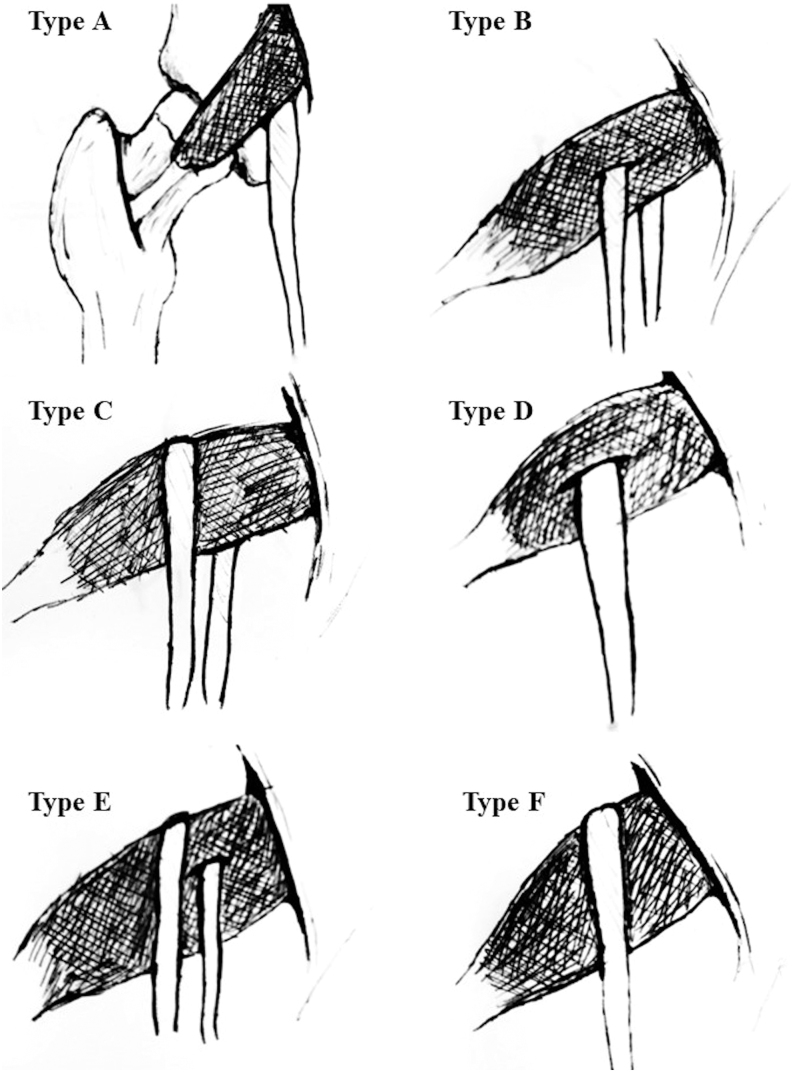
Schematic representation of the first six anatomical relationship types between the sciatic nerve and the PM as described by Beaton and Anson. In the original study of Beaton and Anson, types V and VI were only described hypothetically; the most described type was the type I. The sciatic nerve (A) passes undivided anterior and below PM (type I), (B) is divided into the common peroneal nerve that pierces a bifid PM and the tibial nerve that usually passes anterior and below PM (type II), (C) is divided into the common peroneal and tibial nerve passing one anterior and the other posterior to PM (type III), (D) passes undivided through a bifid PM (type IV), (E) is divided into the common peroneal and tibial nerves passing one through and the other posterior to PM (type V), (F) passes undivided posterior to PM (type VI).

The PM is closely related to the sciatic nerve. Typically, the sciatic nerve splits into its final branches, the tibial and fibular, at the popliteal fossa level. However, in 12% of cases, the nerve is divided proximally at the PM level (27). Beaton and Anson were the first to describe six anatomical relationship types between the sciatic nerve and the PM (28) (Fig. 4). A cadaveric study of 294 limbs supported that type I was the most common anatomical variation while type II was observed in 12 specimens (29). In three of these cases, type II occurred bilaterally. The study also identified rare anatomical variations not classified previously, including a three-headed piriformis, a bilateral supernumerary muscle in the suprapiriform foramen and an inferior gluteal vein passing through a tibial nerve branch (29).

Two systematic reviews also demonstrated that type II is the most common anatomical variation after the type I typical configuration (Fig. 4 and Table 3). If a patient has an anatomical variation on one side, it is more likely to have the same or different variation in the contralateral field (30). Type II is twice as common in females as males and is more prevalent in East Asia than in other areas (30). A case report highlighted a unique case where the ventral rami below the PM formed the sciatic nerve trunk, unlike the previously described cases (31). According to Pecina, in 16.15% of type II variations, the sciatic nerve’s common peroneal part passes between two parts of a divided PM. In 4.6% of type II cases, the branch pierces the PM and remains undivided (32).

**Table 3:** Studies evaluating groups of patients and reporting the number of hips belonging to the six categories according to Beaton & Anson (28). Results are presented as percentages.

Study	Hips, *n*	Type I	Type II	Type III	Type IV	Type V	Type VI	Other variations
Beaton & Anson (28)	240	84.20	11.7	3.3	0.8	-	-	-
Natsis *et al.* (29)	294	93.60	4.1	0.30	0.30	-	0.30	1.40
Pecina (32)	130	78.46	20.77	0.77	-	-	-	-
Pokorny *et al.* (33)	182	79.10	-	-	14.30	2.20	4.40	-
Benzon *et al.* (34)	66	98.50	1.50	-	-	-	-	-
Varenika *et al.* (35)	643	86.80	12.90	-	-	-	-	-
Smoll *et al.* (36)	6,062	83.1	13.7	1.29	0.53	0.08	0.0	-
Poutoglidou *et al.* (30)	1,612	83–90	5–10	0–3	0–2	-	-	-

### Blood supply and innervation

The PM receives blood from the superior and inferior gluteal arteries and the internal pudendal artery (37, 38). Nerve supply to the PM is supported from the fifth lumbar (L5) to the third sacral (S3) ventral rami with the S1 and S2 ventral rami being the most common nerve supply (39). A recent study showed that the PM usually receives nerve supply from more than one nerve, supporting the PM as having a variable nerve supply source (39). Iwanaga *et al.* studied bilaterally ten fresh-frozen cadavers evaluating the nerve branches to the PM (39). The PM was innervated by two to three nerves on 80% of the sides, mainly from the superior gluteal nerve and the S1 and S2 ventral rami. A smaller percentage of branches came from the inferior gluteal nerve and the L5 ventral ramus. Occasionally, the PM is innervated only by the S2 (1). Another study supported that L5, S1 and S2 spinal nerve branches innervate the PM from the sciatic nerve (40).

Russel *et al.* evaluated the relationship between PM and the sacral nerve roots on T1-weighted routine MRI in 100 sequential patients (22). They reported that the S1 nerve roots were located above the muscle in 99.5% and S4 nerve roots below the PM in 95% of the cases. Seventy-five percent of the S2 and 97% of the S3 nerve roots transverse the muscle primarily in the superior and the inferior half respectively (22). Due to the PM nerve supply, muscle function may be affected by lumbar disc herniation. A recent case report reported an L5-S1 paracentral disc herniation in a 29-year-old girl with sciatica, compressing the descending S1 nerve root and leading to PM atrophy. The high incidence of lower lumbar disc herniation highlights the need to evaluate them when assessing the PM function (41).

### Function

The primary function of PM is to externally rotate the hip, working alongside other SHERs (1, 18, 42). However, the hip position impacts the PM functions. According to Cass (43), the PM’s primary function is external hip rotation when the leg is extended. However, it also serves as a weak thigh abductor when flexing the hip (40). PM functions are essential for maintaining proper posture, stabilizing the body in a standing or walking position and preventing falls by shifting the body weight to the opposite side. As a result, the PM serves multiple roles as an external rotator and abductor (38), weak hip flexor (44) and stabilizer (45).

In addition, the PM works with the ipsilateral gluteus maximus to tilt the sacrum anteriorly and rotate it to the opposite side (46). PM tightness can lead to coccygodynia. The PM is also responsible for limiting the femoral head backward movement while the hip is being flexed. This occurs because the muscle shifts to a more posterior position about the hip joint during hip flexion and stretches when the hip is adducted, internally rotated and flexed, reducing the space between the sacrotuberous ligament, the inferior PM border and the superior gemellus (7).

During the stance phase of walking, the hip rotates from external to internal, causing the PM to lengthen and stretch due to the pelvis dropping on the shorter leg side. In the swing phase, a second contraction occurs with external rotation, resulting in a double PM exercise. Consequently, PM may cause hypertrophy, pressing the sciatic nerve and causing severe pain radiating down the leg (47, 48).

### PM-preserving posterior hip approaches

Several studies have investigated the outcome of sparing the PM alone or with the SHERs and releasing then repairing obturator externus and recently inferior gemellus (5, 6, 49, 50, 51, 52, 53, 54, 55, 56, 57, 58). Table 4 maps and summarizes the most representative clinical PM-preserving clinical studies so far. Preservation is superior to reattachment in terms of contiguity and muscle atrophy, as found in an MRI study (59), and they can be better preserved through a posterior approach (60). In an early report by Moussallem *et al.* 2012, in a case series of 226 THAs, no dislocation was found when sparing the PM (49). In the same year, a randomized controlled trial of 100 cases randomized to piriformis sparing or standard posterior approach found short-term benefits in improved walk test and patient satisfaction; however, surgeon perception of the approach was more challenging, particularly in obese patients (50). However, a comparative study by Tan *et al.* 2020 found the same long-term functional benefit (52). This is also reflected in a report regarding similar long-term functional benefits this year, but there is reduced blood loss and improved pain control in the short term (56). More recent studies report functional advantages when the piriformis is preserved for hip hemiarthroplasty (57, 58) with SHERs and reattaching obturator externus (54) and piriformis alone for THA (6).

**Table 4:** Studies evaluating PM-preserving posterior approaches compared to standard or lateral approaches.

Study	Year	Study design	Pts, *n*	Outcome	Conclusion
Moussallem *et al.* (49)	2012	CS	226 THAs	No cases of dislocation after 3 years f.u.	Preserving PM is superior to repairing to reduce THA dislocation risk
Khan *et al.* (50)	2012	RCT*	100 THAs	Better 6 min walk test at 2 weeks and better satisfaction at 6 weeks in PM sparing group	Piriformis sparing approach can be more challenging and benefits are short term
Hanly *et al.* (5)	2017	TR	-	The only tendon released is obturator externus. PM and SERs are maintained	Hip function, stability and gait are potential advantages
Siddappa *et al.* (51)	2020	CS	150 THAs	No dislocation (minimum f.u. of 6 months)	May cause less visualization but reduces dislocation risk
Tan *et al.* (52)	2020	RCT*	100 THAs	Same long-term (10-year) functional benefit. Improved muscle volume and grade for PM-sparing	Less injurious to PM. Same long-term function
Wang *et al.* (53)	2021	PCS*	126 THAs	Lower blood loss, hospital stay, time to mobilization, stair use and transfusion in SHER-sparing group	SHER-sparing approach reduces blood loss and pain, confers functional advantage, and improves stability
Charity *et al.* (54)	2023	RCS^†^	285 HAs	Return to pre-injury level odds higher than lateral approach at 3.5 years	Safe for hip HA and returns to pre-injury mobility level better than lateral approach
Selvaratnam *et al.* (55)	2023	CR	1	Modification of SPAIRE by also releasing inferior gemellus to avoid damage	Greater stability than just preserving PM
Wu *et al.* (56)	2023	PRS	200	HHS score similar at 12 months. VAS score lower at 48 h for PM-sparing than conventional	Faster recovery, earlier pain relief but similar functional scores at 12 months
Kenanidis *et al.* (6)	2023	HCCS	400^‡^	STAR had significantly lower mean incision length, hospital stay and higher functional scores early postoperatively	STAR approach associated earlier functional improvement, shorter hospital stay and less transfusion requirement
Viberg *et al.* (57)	2023	HCCS	527 HAs*	PM-sparing resulted in a 50% reduction in dislocation and reoperation rates	PM-sparing was easily introduced and may enable further lowering of dislocation rates
Apinyankul *et al.* (58)	2023	PC*	321 HAs	Lower dislocation rate, mortality and higher functional outcomes in the PM-sparing group	PM preservation was associated with lower dislocation rate mortality and higher functional scores

CS, case series; PCS, prospective comparative study; PC, prospective cohort; PRS, prospective randomized study; CR, case report; HCCS, historical cohort comparison study; RCS, retrospective comparative study; TR, technical report; PM, piriformis; THA, total hip arthroplasty; f.u., follow-up; SHER, short hip external rotators; SPAIRE, Spare Piriformis and Internus, Repair Externus; DSA, direct superior approach; STAR, Superior Transverse Atraumatic Reconstruction; HA, hemiarthroplasty.

* PM-sparing vs standard posterior.

^†^ SPAIRE vs lateral.

^‡^ 200 DSA vs 200 STAR.

## Discussion

This scoping review examines the diverse PM anatomy and function, its relationship with adjacent structures and the main outcomes of the existing literature on PM-preserving THA clinical studies. The review emphasizes the PM’s topography, anatomy and its relationship with other SHERs and the sciatic nerve. This aids the surgeon in locating and preserving the PM and understanding the anatomical structures at risk during preservation. The ultimate objective is to offer an overview of the PM’s involvement in hip stability and surgical anatomy and summarize the literature on the outcomes of clinical studies on THA-preserving PM to improve readers’ understanding and identify areas for further research.

Knowing the high variability of the anatomy and the relationship of the muscle with the surrounding structures is crucial to preserving the muscle safely. PM identification is not always straightforward and easy and preservation is not always feasible. Formerly, it was believed that the PM muscle attaches to the femur at the piriformis fossa, making its insertion point susceptible during the femoral intramedullary nailing (13). However, modern research indicates that the PM insertion point is higher and on the GT medial side, away from the typical site of the GT osteotomy (13). Identifying the muscle’s insertion point can be challenging, particularly when the distal PM tendon merges with the other SHER tendons. Surgeons usually use the muscle’s orientation relative to the other SHERs as the prominent landmark to locate the PM tendon. The PM muscle generally comes at a 45-degree angle to the GT while the other SHERs are oriented more vertically to the femur (Fig. 2).

The PM plays a crucial role in hip movement and contributes to postural stability during standing and walking (42) and prevents the posterior translocation of the femoral head during hip flexion (7). Knowing the PM’s nerve supply is essential to understand the relationship of PM with other pathologies. Unlike other muscles, the PM does not rely on a single nerve for innervation (39). The primary nerve supply comes from the S1 and S2 ventral rami while the L5 to S3 ventral rami may also contribute to nerve supply (39, 40). A link exists between L5 and S1 disc herniation and PM pathology and function. If the L5 nerve root is compressed, it can lead to progressive atrophy of the PM, ultimately affecting its function, hip stability and overall body posture.

The relationship between the PM and the sciatic nerve is highly significant for surgeons, as it helps them understand the structures at risk during PM preservation. Typically, the sciatic nerve passes undivided anterior and below PM and splits into its final two branches at the level of the popliteal fossa (27). Numerous studies have identified anatomical variations in the relationship between the PM and sciatic nerve (29). Surgeons who approach the posterior hip must be mindful of this muscle variability and the variable relation to the sciatic nerve, particularly when attempting to perform minimally invasive or muscle-sparing approaches for THA.

The PM muscle is a vital component of hip anatomy (61, 62). It is commonly identified during posterior THA approaches as it helps locate the posterior thylacus and grants access to the hip region. One of the latest muscle-sparing procedures is the direct superior approach (DSA), which involves performing a tenotomy on the PM and OI tendons near their femoral insertion to reach the hip joint (63). However, these tendons are meticulously reattached via transosseous channels in the GT post-surgery to restore PM function. The technical differences between the standard posterior and DSA procedures have been extensively studied in a comprehensive study encompassing 175,543 primary THAs from the Dutch Arthroplasty Register, with preliminary results suggesting that using the DSA during THA may reduce the revision risk due to dislocation (64). Despite this, insufficient evidence supports an overall reduction in revision risk compared to the standard posterior lateral approach. Nonetheless, a different study has shown that the DSA approach may lead to advanced functional recovery and shorter hospital stays for patients undergoing THA compared to the standard posterior hip approach (65).

**Figure 5:**
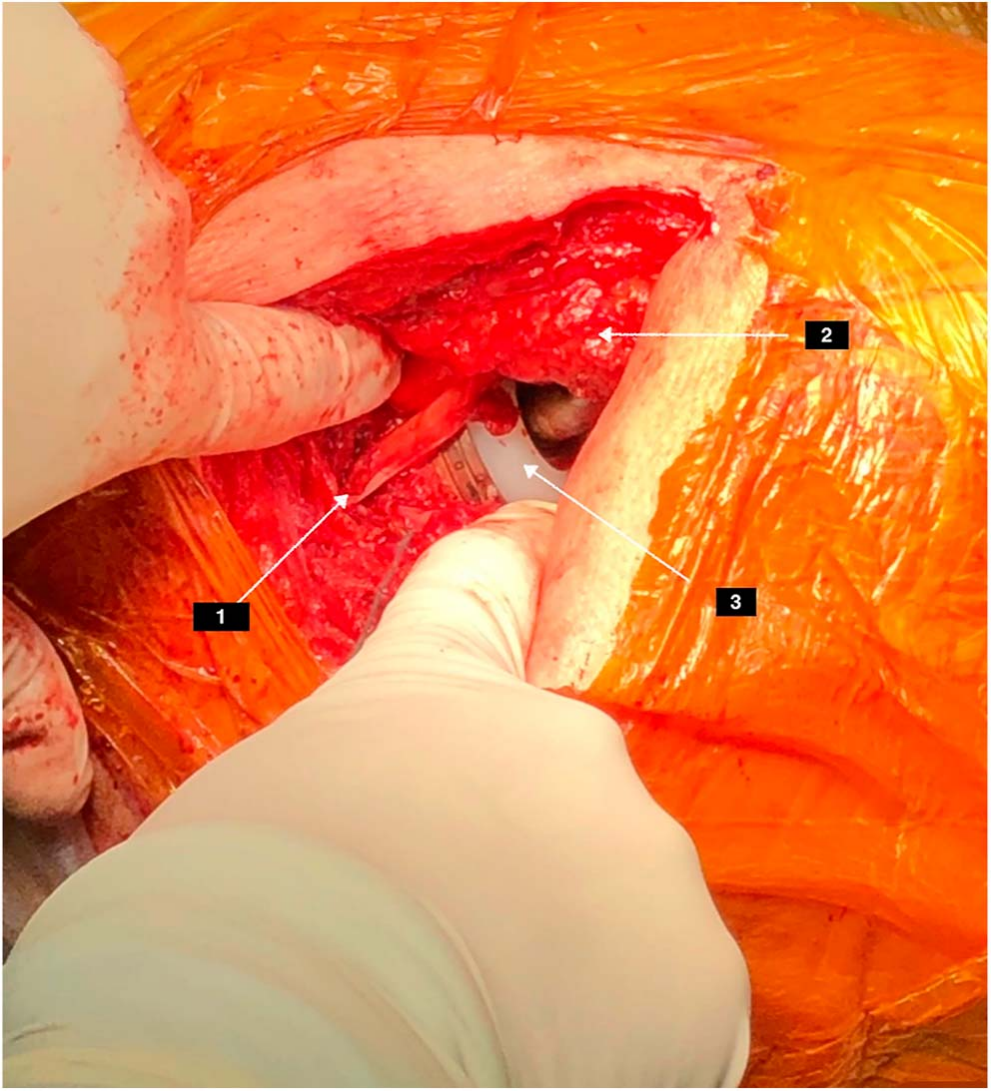
Intraoperative THA picture through the piriformis-preserving mini-posterior STAR (Superior Transverse Atraumatic Reconstruction) approach showing 1. PM 2. major trochanter 3. acetabular insert.

Advancements in understanding the PM function and its role in hip joint stability have resulted in the development of muscle-sparing hip approaches such as the SPAIRE (Spare Piriformis and Internus, Repair Externus) (5) and the STAR (Superior Transverse Atraumatic Reconstruction) (6), which prioritize the PM preservation (Fig. 5). Studies on these piriformis-sparing techniques have shown a decreased risk of PM injury and similar long-term functional benefits to the standard posterior approach even after a decade of follow-up (52). Moreover, the SPAIRE technique has displayed a favorable safety profile in hip hemiarthroplasty and has the potential to maintain pre-injury mobility levels better when compared to the conventional lateral approach (54). These findings underscore the PM significance and emphasize the need for further research into muscle-preserving approaches.

Our study has some acknowledged limitations that should be taken into consideration. Instead of conducting a systematic review, we opted for a scoping review approach to present the relevant bibliography on the PM. While this approach provides a broad overview, it may lead to potential biases due to less stringent inclusion criteria. Furthermore, the lack of a formal quality assessment of the studies included may impact the overall strength of our research. Nevertheless, this scoping review proves valuable in highlighting the primary areas of the literature related to our research topic and consolidating information on the PM. It lays the groundwork for future research and may pave the way for more comprehensive systematic reviews.

## Conclusion

The anatomy of the PM muscle and its relationship to surrounding structures can vary significantly. Preserving the PM muscle during hip surgery is not always straightforward and easy and can pose challenges. Understanding the anatomical variations of PM muscle can help surgeons preserve the muscle safely. There is a limited amount of the literature on clinical studies related to PM-preserving THA and hemiarthroplasty. While some studies suggest potential benefits in terms of hip stability, reduced dislocation risk and improved functional outcomes in the short term, further high-level research is necessary.

## ICMJE Statement of Interest

The authors declare that there is no conflict of interest that could be perceived as prejudicing the impartiality of the work reported.

## Funding Statement

This research did not receive any specific grant from funding agencies in the public, commercial or not-for-profit sectors.

## Author contribution statement

The authors have full control of primary data and agree to allow the journal to review these data upon request.
